# Effect of Silicone Inlaid Materials on Reinforcing Compressive Strength of Weft-Knitted Spacer Fabric for Cushioning Applications

**DOI:** 10.3390/polym13213645

**Published:** 2021-10-22

**Authors:** Annie Yu, Sachiko Sukigara, Miwa Shirakihara

**Affiliations:** Faculty of Fiber Science and Engineering, Kyoto Institute of Technology, Matsugasaki, Sakyo-ku, Kyoto 606-8585, Japan; sukigara@kit.ac.jp (S.S.); m0651009@edu.kit.ac.jp (M.S.)

**Keywords:** weft-knitted spacer fabric, cushioning, compression, tensile strength, silicone inlay, sandwich textile structure

## Abstract

Spacer fabrics are commonly used as cushioning materials. They can be reinforced by using a knitting method to inlay materials into the connective layer which reinforces the structure of the fabric. The compression properties of three samples that were fabricated by inlaying three different types of silicone-based elastic tubes and one sample without inlaid material have been investigated. The mechanical properties of the elastic tubes were evaluated and their relationship to the compression properties of the inlaid spacer fabrics was analysed. The compression behaviour of the spacer fabrics at an initial compressive strain of 10% is not affected by the presence of the inlaid tubes. The Young’s modulus of the inlaid tubes shows a correlation with fabric compression. Amongst the inlaid fabric samples, the spacer fabric inlaid with highly elastic silicone foam tubes can absorb more compression energy, while that inlaid with silicone tubes of higher tensile strength has higher compressive stiffness.

## 1. Introduction

Cushioning materials can be found in many different types of apparel and wearable items that provide shock absorption and wearer protection. Traditionally, polymeric foams are used in insoles, bra cups, and protective apparel to deliver the cushioning function [1,2,3]. Recently, spacer fabrics which are weft or warp knitted have been used as an alternative to foam materials. They are now a viable option because they have a unique three-dimensional (3D) knitted structure and provide the products with higher air and water vapour permeabilities and breathability [4,5,6,7,8]. Spacer fabrics can also be readily found in daily life items, such as in shoes, chairs, car seats, carpets, mattresses, backpacks, etc. [9].

Compression behaviour is an important criterion for determining whether or not a material can offer suitable cushioning functions and hand feel for different end-uses. In terms of polymeric foam, the compression properties can be controlled by varying the composition and the density to accommodate various applications [10,11]. Spacer fabric consists of two surface layers that are connected by spacer yarns which form a connective layer. Variations in any component of spacer fabric can affect its mechanical properties and wear comfort. The compression properties of spacer fabrics have often been studied. The elasticity of yarns used in the surface layer is one of the factors that contributes to the compression properties [12,13,14]. The compression properties of weft-knitted spacer fabric have been found to be affected by the number of tuck stitches in the surface layers [15]. However, it has also been shown that the compression properties of spacer fabric are related to the connective layer [16,17,18]. Monofilament and multifilament yarns are commonly used as spacer yarns and can impart entirely different properties to the fabric [19,20,21]. The thickness, composition, connecting distance, inclination angle, and pattern of the spacer yarns can be used to control the thickness and compressive stiffness of the spacer fabric [22,23,24]. The previous studies related to compression behaviour of spacer fabrics mainly focus on the structural properties. However, it can be challenging to produce a thin fabric with high compression strength and high energy absorption when using a conventional spacer fabric structure. A spacer fabric that is thin enough for use as insoles or protective garments (less than 1 cm in thickness) could easily collapse from stress produced by the human body [25]. However, if a more compact connective layer is used, the spacer fabric becomes heavy and stiff, thus reducing its cushioning ability. Hamedi et al., proposed the use of nickel–titanium alloy wires as the spacer yarn [26]. The spacer fabric shows an improvement on the compression energy absorption; however, the cost of the fabric is also largely increased. Moreover, investigation in applying materials other than polyester or polyamide filament yarns in the connective layer of the spacer fabric is still limited. 

In this study, a composite structure that consists of additional silicone-based materials was investigated so as to enhance the cushioning properties of spacer fabric. A novel sandwich structure with inlaid silicone tubes in the connective layer of spacer fabric has previously been developed [27]. Silicone is a synthetic polymer with a silicon–oxygen main chain [28]. Silicone is flexible, flame retardant, and relatively inert. Silicone inlaid tubes offer extra support to reinforce the spacer structure so that the fabric can withstand pre-stress from the body during application without the flexibility and energy absorption properties being sacrificed. In order to further investigate the effect of inlaid materials on the properties of spacer fabric, samples made with three different kinds of silicone-based tubular materials were fabricated and evaluated. The main purpose was to understand the relationship between the mechanical properties of the inlaid tubular materials and the compression properties of spacer fabric. The findings can contribute to furthering the development of sandwich structured textile materials and enhance wearable cushioning products. The inlaid materials can become a new parameter in adjusting the compression and cushioning properties of knitted spacer fabrics which allows the fabric to provide the desired energy absorption ability for various end-uses.

## 2. Materials and Methodology

### 2.1. Materials

The yarns for knitting the surface layers of the spacer fabric samples included 450D 3-ply 100% polyester drawn textured yarn (LS 1/20, Amossa, Osaka, Japan) and 140D spandex yarn (Heng Jing Limit, Jiangsu, China). The spacer yarn was 100% polyester monofilaments with a diameter of 0.12 mm (Nantong Ntec Monofilament Technology Co., Ltd., Nantong, China). Three types of silicone-based tubular materials, including silicone foam rods, silicone rods, and silicone hollow tubes (Yuema, Shanghai, China) were inlaid in the connective layer of the spacer fabrics, as listed in Table 1. The three types of inlay materials have a similar thickness but a different linear density. T1, which incorporates silicone foam as the tubular material, is relatively light in weight. The good elasticity and low density of silicone foams make it suitable to be used in challenging application such as shock absorbers, wound dressings, and joint sealants [29,30]. T2 and T3 both incorporate silicone rubber. Silicone rubbers can be made into tubes, hose, gaskets, and seals [31]. T2 incorporate silicone in a solid rod form, while T3 is a hollow tube form.

### 2.2. Preparation of the Inlaid Spacer Fabric

**Figure 1:**
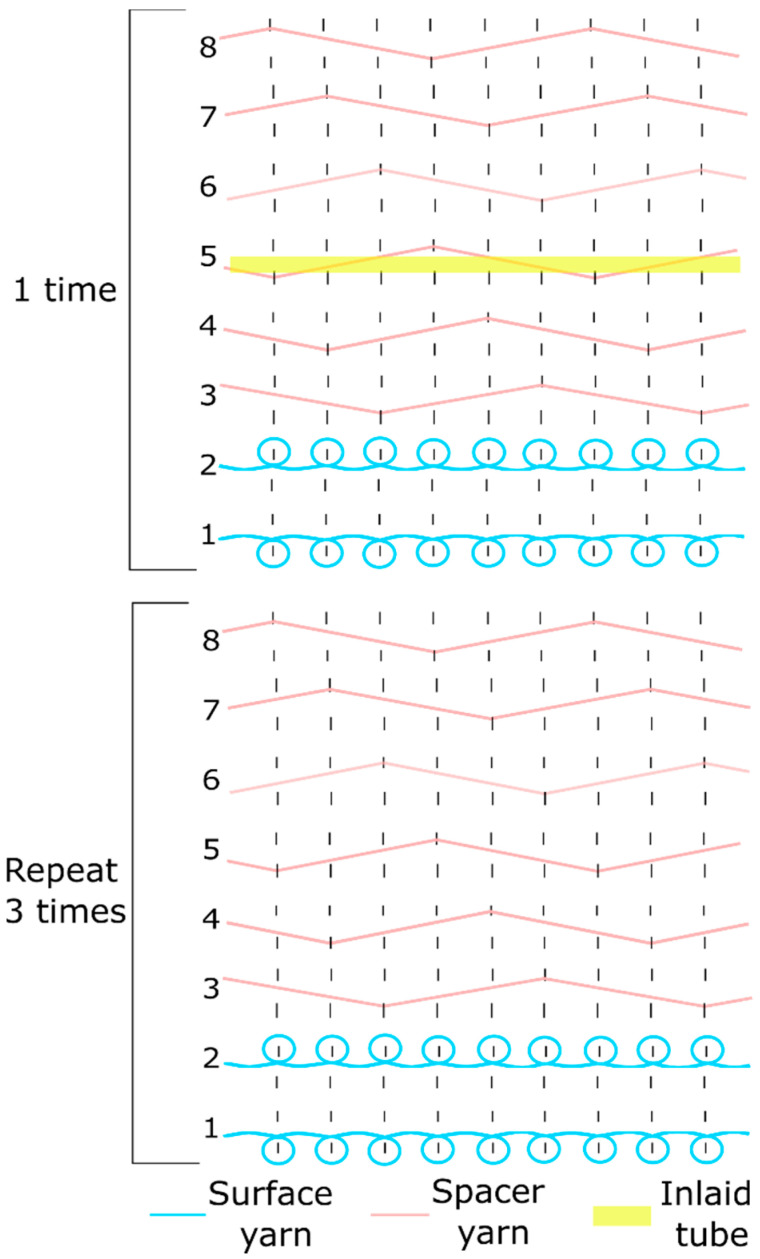
Yarn path diagram of the spacer fabric samples with inlaid tubes.

Three samples inlaid with each type of tubular material and one sample without any inlaid material were produced by using a 10-gauge v-bed flat knitting machine (SWG091N210G, Shima Seiki, Wakayama, Japan). The two surface layers were knitted with a single jersey structure and the connective layer was a spacer structure with a linking distance of 6 needles for all the samples. One course of the spacer fabric consisted of 2 courses of knit stitches on the surface and 6 tuck stitch courses of spacer yarn. Following a previous study, the tubular materials were inlaid into the connective layer with miss stitches [27]. Therefore, the tubular materials did not come into contact with the knitting needles and floated between the front and back needle beds. The inlaid course was carried out between the tuck courses of spacer yarn and hence the spacer yarns acted as a net to hold the silicone tubes in place. One course of inlay was inserted in every 4th knitting course of the spacer fabric (Figure 1). The weight, thickness, and cross-sectional views of the four fabric samples are shown in Table 2.

### 2.3. Evaluation of Mechanical Properties of Inlaid Tubular Materials

**Figure 2:**
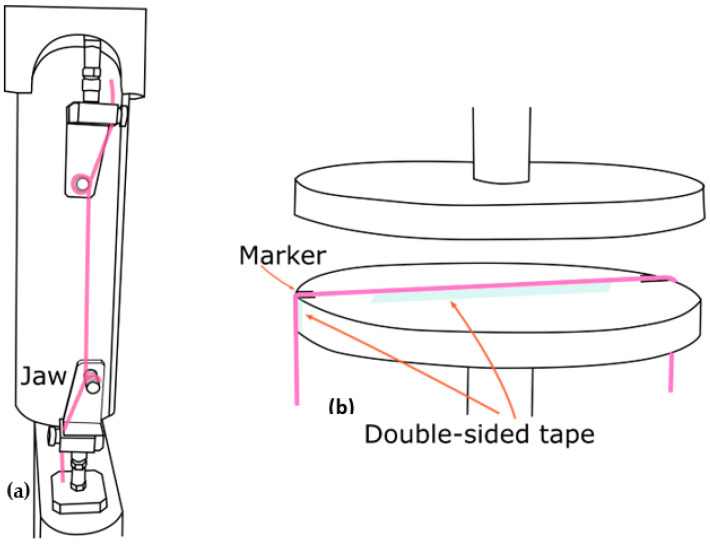
Setting of (**a**) tensile test and (**b**) compression test of the tubular samples.

Tensile and compression tests were conducted on the three tubular samples by using a universal testing machine (EZ-S, Shimadzu, Kyoto, Japan). The tensile test was conducted in accordance with ASTM D2731-15, the standard test method for elastic properties of elastomeric yarns. The tubular sample was mounted between a pair of jaws (Figure 2a). The gauge length was set at 50 mm with a pre-tension of 2.55 g. The sample was subjected to 5 loading and unloading cycles. The sample was extended at a rate of 500 mm/min, held at the maximum extension limit for 30 s, and returned at a rate of 100 mm/min. The maximum extension was set at 300% of the gauge length. As T1 and T2 failed to extend to the 300% strain, 75% of the elongation at first break was used as the maximum extension instead. Therefore, T1 and T2 were extended up to 202% and 224% of the gauge length, respectively. The compression test was carried out by using a pair of compression plates. The sample was mounted onto the centre of the plate at a length of 118 mm. Double-sided tape was used to hold the sample in place during testing (Figure 2b). The compression speed was 20 mm/min with a maximum compression displacement of 0.6 mm. The samples were conditioned under a standard environment (20 ± 2 °C, 65 ± 2% relative humidity) for 24 h before they were tested. 

### 2.4. Evaluation of Compression Properties of the Spacer Fabrics

A compression test on the fabric samples was carried out by using the same testing machine along with a pair of compression plates with a diameter of 118 mm. The fabric samples were prepared with dimensions of 50 mm × 50 mm. The compression rate was 12 mm/min with a maximum compression stress of 60 kPa. Four specimens of each sample were tested. The compression energy of each sample was calculated as the integral of the compressive loading (WC) and unloading (WC’). All of the fabric samples were allowed to relax for one week after released from the knitting machine and stored in a standard environment (20 ± 2 °C, 65 ± 2% relative humidity) before testing. ANOVA was adopted to analyse the effect of the inlaid materials on the compression strain and compression energy. A Sidak post hoc test was used to analyse the effect between pairs. The alpha level was set at 0.05 for statistical significance.

## 3. Results and Discussion

### 3.1. Analysis of the Tensile Properties of the Inlaid Tubular Materials

**Figure 3:**
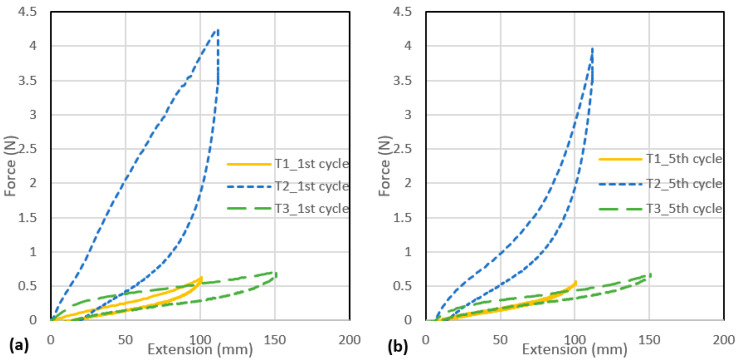

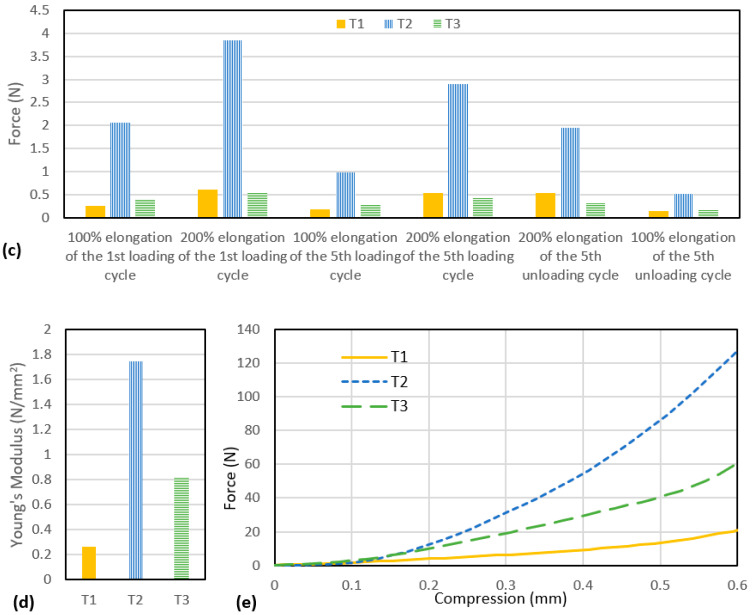
Tensile extension–force curves for (**a**) the first cycle of extension, and (**b**) the fifth cycle of extension; (**c**) force for 100% and 200% elongation; (**d**) Young’s modulus, and (**e**) compression displacement–force curves of the three tubular samples.

The plotted loading and unloading curves of the first and the fifth cycles of the tensile test of the three tubular samples are presented in Figure 3a,b. The force at 100% and 200% elongation of the tubes at the first and fifth loading cycles and the fifth unloading cycle, together with the Young’s modulus measured from the first extension loading are shown in Figure 3c,d. Figure 3e shows the displacement–force curves obtained from the compression test. The three tubular samples show very different non-linear elastic behaviours. Silicone foam is a porous viscoelastic polymer foamed from silicone rubbers [32,33]. Silicone foam has the properties of silicone combined with foam properties, light weight, and good flexibility [34]. T1 is the most elastic and has the lowest Young’s modulus amongst the three tubular samples. A relatively small force is required to extend T1 and the loss in elastic hysteresis is also small. T2 is solid rod form of silicone, has the highest Young’s modulus, requires the largest force for extension, and shows a large hysteresis, especially in the first cycle of extension. T3 is hollow tube form of silicone and therefore has a lower weight and tensile strength than T2. T3 can be extended to the longest length at break. In comparison to T1, T3 requires a slightly higher force of 0.1 N to extend to a strain of 100% but 0.1 N less to extend to a strain of 200%. T2 has the highest compressive stiffness, followed by T3, whereas T1 is the softest material and most easily compressed. By inlaying the three tubular samples which have a different tensile strength, elasticity and stiffness into the spacer fabric, the effect of the mechanical properties of the inlaid materials on fabric compression properties can be identified.

### 3.2. Effect of the Inlaid Tubes on the Compression Behaviour of Spacer Fabric

**Figure 4:**
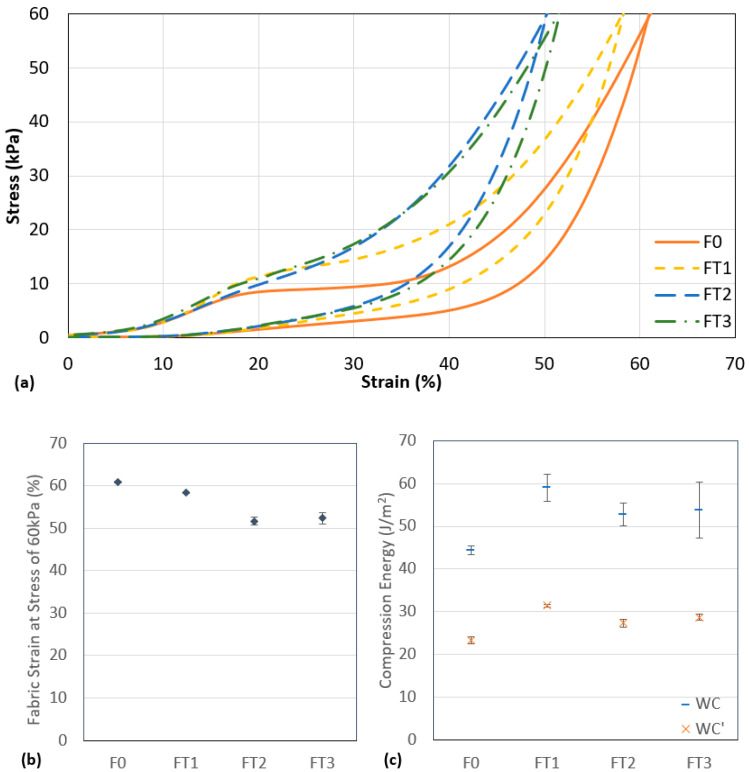
(**a**) Compression stress–strain curve, (**b**) fabric strain at a stress of 60 kPa, and (**c**) compression energy of four fabric samples.

The compression stress–strain curves, fabric strain at a stress of 60 kPa, and the compression energy of the four fabric samples are shown in Figure 4. At a compressive strain of 0 to 10%, the compression behaviour of the four fabrics is very similar because they are constructed with the same surface and connective structures that have the same materials. The initial stress up to 3.5 kPa compresses the loose surface layers and tightens the spacer structure. This shows that the initial softness of the spacer fabric is not affected by the presence of the inlaid tube in the connective layer.

When the compression stress is further increased, the monofilament yarns start to deform and buckle. F0 starts to collapse, thus showing a decrease in the slope of the stress–-strain curve and entering a plateau stage at a stress of 8.5 kPa. In the plateau stage, the monofilament yarns can no longer hold the connective structure which leads to the shearing of the fabric layers and rotation of the yarns. The fabric can be easily compressed with a small increase in stress. FT1, FT2, and FT3 consist of inlaid tubes to give extra support to the structure and withstand some of the stress applied. As shown in Figure 4a, the inlaid spacer fabric can withstand a higher compression stress than the one without inlay. This supports that inlaid yarns decrease the deformation ability of knitted fabrics [35]. The different inlaid tubes have different Young’s moduli and mechanical properties and thus different compression properties can be found for all three fabrics. FT1 reaches the plateau stage at 11.1 kPa of compression. The plateau stage of FT1 covers a smaller range of strain when compared with F0. This is because the inlaid silicone foam rods act as a buffer to absorb a certain amount of the compression energy applied to the connective layer. On the other hand, there is no prominent plateau stage for FT2 and FT3. The inlaid tubes T2 and T3 are relatively stiff and can withstand most of the compression stress that acts on the connective layer and exceed the energy absorption capacity of the monofilament yarns. Therefore, the plateau stage that typically appears in the compression curve of spacer fabric is not shown for FT2 and FT3.

Significant differences (*p* < 0.05) between the four samples on the fabric strain at a stress of 60 kPa, WC, and WC’ are found in the results of ANOVA. For the fabric strain at 60 kPa, F0 and F1 show significant difference with all the other samples while there is no significant difference between F2 and F3. At a stress of 60 kPa, F0 was compressed to the highest strain of 61%. With the silicone foam rod inlay, the strain at 60 kPa of stress for FT1 decreases by 58%. The fabric structure of FT2 and FT3 is supported by the relatively stiffer inlaid tubes and hence even smaller strains are shown at a stress of 60 kPa. Moreover, the spacer fabrics with inlay have a significantly higher WC than conventional spacer fabric with no inlay. This shows that the inlaid tubes could help to absorb more compression energy than regular spacer fabric. The inlaid structure can provide better support against impact forces when used as padding or cushioning materials. Although no significant difference (*p* > 0.05) was shown on the WC between the pairwise comparison of the three spacer fabrics with inlay, FT1 shows a significant difference with FT2 and FT3 on the WC’. The compression behaviour and the compression energy of the spacer fabric can be affected by the inlaid material used. 

### 3.3. Relationship between Properties of Inlaid Tubes and Spacer Fabric Properties

**Figure 5:**
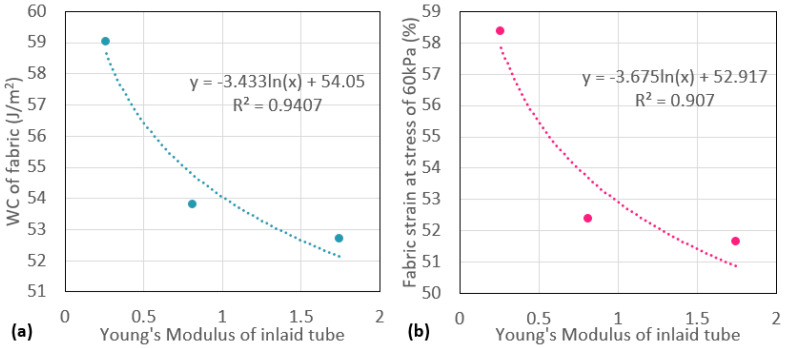
Relationship between the Young’s modulus of the inlaid tube, (**a**) the compression energy of the fabric samples, and (**b**) the fabric strain at a stress of 60 kPa.

The relationship between the elasticity of the inlaid tubes and the compression properties of the spacer fabric was further investigated. In Figure 5, the logarithmic relationships between the Young’s modulus of the inlaid tube samples with the WC of the spacer fabric samples and the fabric strain at a stress of 60 kPa show a high coefficient of determination (R2 > 0.9). The inlaid tubular materials show a significant effect on the compression behaviour of the spacer fabric. Amongst the three types of inlaid spacer fabrics, FT1 shows the highest WC. By inlaying a softer and higher elastic material such as the silicone foam rods, the spacer fabric can absorb a larger amount of compression energy. T2 is however heavy and has high tensile strength. Therefore, FT2 is stiffer than FT1 and FT3, especially when the compressive strain is above 35%, where the monofilament yarns have buckled and the inlaid tubes mainly support the fabric against compression forces. The fabric compression behaviour of FT2 and FT3 is similar. The hollow silicone tube, T3, has a Young’s modulus and compressive stiffness that ranges between those of T1 and T2. Therefore, FT3 has a slightly higher WC than FT2. As only three samples are studied, it is difficult to generalise the results to all the different types of inlay materials and inlaid spacer fabric. However, the correlation between tensile properties of the silicone foam rod, silicone rod, and silicone hollow tube used in this study and the corresponding inlaid spacer fabric is observed. The mechanical properties of the inlaid materials can affect the compression properties of the inlaid spacer fabric.

## 4. Conclusions

The effect of inlaying tubular materials in the connective layer of spacer fabric on compression reinforcement has been investigated. Three weft-knitted spacer fabric samples inlaid with different tubular materials and one conventional spacer fabric without inlaid material as the reference were fabricated. The mechanical properties of the tubular samples and the compression properties of the fabric samples and the relationship between them were evaluated. The following conclusions were made based on the findings:

- The compression behaviour of the spacer fabric at an initial compressive strain of 10% is not affected by the presence of inlaid tubes in the connective layer.
- The inlaid spacer fabrics require higher stress to enter the plateau stage than the conventional spacer fabric. When an inlaid material with higher tensile strength and compression strength is used, no obvious plateau stage can be found in the compression stress–strain curves of the fabric.
- The inlaid spacer fabrics not only have higher compression strength but can also absorb more compression energy than the conventional spacer fabric. The inlaying of elastic materials such as silicone foam or silicone rods effectively reinforces the spacer fabric.
- Different inlay materials with different Young’s moduli and tensile behaviours can affect the compression energy and stiffness of the resultant fabrics. The spacer fabric inlaid with silicone foam rods, which have lower tensile strength and compression strength than silicone rods and silicone hollow tubes, can absorb more compression energy. On the other hand, the spacer fabric that is inlaid with silicone rods with a high tensile strength and compression strength has the highest compressive stiffness amongst the fabric samples.

A better understanding of the effect of different types of inlaid tubes on the compression properties of weft-knitted spacer fabric is provided. The findings can be used as a reference in the design and development of spacer fabrics to meet the requirements of various cushioning applications.

## Figures and Tables

**Table 1:** Details of the three tubular samples.

	Type	Diameter (mm)	Weight (g/m)	Longitudinal View	Cross-Sectional View
T1	Silicone foam rod	1.23	0.8	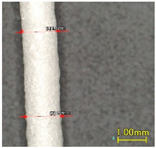	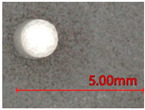
T2	Silicone rod	1.22	1.4	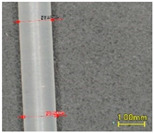	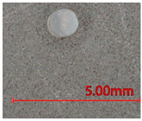
T3	Silicone hollow tube	1.12	1.0	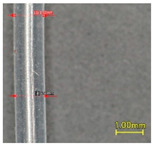	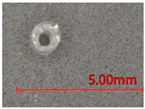

**Table 2:** Details of spacer fabric samples.

Fabric Sample	Inlay	Weight (kg/m^2^)		Thickness (mm)		Cross-Sectional View	
						Course-Wise	Wale-Wise
F0	No inlay	468	±4.3	4.41	±0.13	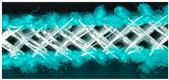	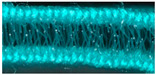
FT1	Inlaid with T1	681	±19.8	5.26	±0.03	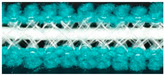	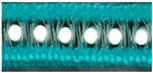
FT2	Inlaid with T2	865	±23.2	5.28	±0.13	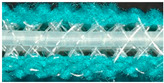	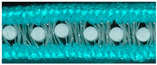
FT3	Inlaid with T3	784	±14.8	5.43	±0.06	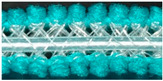	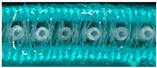

## Data Availability

Not applicable.
